# Pediatrician and parental evaluation of child neurodevelopment at 2 years of age

**DOI:** 10.1186/s12887-024-04616-2

**Published:** 2024-02-22

**Authors:** Giulia Segre, Antonio Clavenna, Elisa Roberti, Francesca Scarpellini, Massimo Cartabia, Chiara Pandolfini, Valeria Tessarollo, Ilaria Costantino, Maurizio Bonati

**Affiliations:** 1 Laboratory of Epidemiology of Developing Age, Department of Medical Epidemiology, Istituto di Ricerche Farmacologiche Mario Negri IRCCS, Via Mario Negri 2, Milan, 20156 Italy; 2 Child Neuropsychiatry Unit, Department of Health Sciences, San Paolo Hospital, ASST Santi Paolo e Carlo, Università degli Studi di Milano, Milan, Italy; 3 Centro Psicodiagnostico Italiano, Milan, Italy

**Keywords:** Child development, Child health, Neurodevelopmental disorders, Primary health care, Cohort study

## Abstract

**Background:**

The early identification of infants with a risk for neurodevelopmental disorders in the first few years of life is essential for better developmental outcomes. Screenings should be carried out by combining the family pediatricians’ and parents’ perspectives, the two fundamental sources of information on children’s health. The present study has three aims: (a) to test the feasibility of parent-report instruments to detect warning signs in their children’s development; (b) to ascertain whether there is an agreement between the family pediatricians’ (FP) clinical judgments of warning signs and the parental perceptions; (c) to determine whether there is a link between parents’ distress and child development.

**Methods:**

Within the NASCITA birth cohort, in addition to the family pediatrician’s clinical evaluation with routine tools, the Modified Checklist for Autism in Toddlers, Revised (M-CHAT-R) was completed by parents to assess the child’s language, social skills, behavior, and sensory areas. Parents were also asked to complete the Parenting Stress Index, Short Form (PSI-SF) to verify the magnitude of stress in the parent-child system. Univariate and multivariate analyses were performed to evaluate the association between child and parental characteristics and the presence of warning signs.

**Results:**

The follow-up assessment was completed for 435 infants: 69 (15.8%) presented warning signs: 43 in the pediatrician’s assessment and 36 in the M-CHAT-R (10 in both). A total of 16 children (14 with warning signs) received a diagnosis after a specialist evaluation. Being male (OR 2.46, 95%CI: 1.23–4.91) and having sleep disorders (OR 2.43, 95% CI 1.17–5.04) was associated with a greater likelihood of warning signs in the multivariate analysis, while reading aloud was a protective factor (not exposed versus exposed (OR = 3.14; 95% CI 1.60–6.17). For 73 children (18.4%), at least one parent tested positive for PSI-SF. An increased prevalence of parental distress was observed in children with warning signs (OR 2.36, 95% CI 1.27–4.37).

**Conclusions:**

Integrating physician and parental perspectives during well-child visits and in clinical practice appears feasible and can improve the identification of children at risk of developmental disorders.

**Supplementary Information:**

The online version contains supplementary material available at 10.1186/s12887-024-04616-2.

## Background

In the first few years of life, children reach numerous developmental milestones in various domains, acquiring cognitive, social, and emotional skills that will provide the foundations for their lifelong health and achievements [1, 2]. At the same time, exposure to environmental stressors can have negative long-term consequences for children’s early development [3]. Early interventions are crucial to prevent motor, cognitive, and emotional impairments.

Nowadays, there is an increase in formalized developmental screenings in primary healthcare towards detecting developmental disorders earlier [4]. The American Academy of Pediatrics [5] recommends screenings at different age stages, with attention to postpartum depression in mothers during the first year of life [6], to the identification of developmental disorders at 9–30 months, and the early identification of autism at 18–24 months [7]. Pediatricians are the first healthcare providers to advocate for infants and children’s health and safety [8–10]. They monitor development and consider all aspects of a child’s well-being, including biological, social, and psychological factors [10]. There has been increasing effort to identify and intervene early in children with developmental disabilities or delays because it has been demonstrated that delayed diagnoses lead to missed opportunities for interventions known to decrease lifetime costs, reduce the chances of future developmental disorders, and prevent secondary sequelae [11, 12]. In monitoring neurobehavioral development, it is therefore fundamental to consider the shared perspectives of parents and pediatricians. Parents are the infants’ primary care environment and through clinical encounters with families, pediatricians can access all aspects of a child’s well-being, including biological, social, and psychological factors. The World Health Organization encourages establishing a close relationship between parents and healthcare providers in identifying infants with a risk for neurodevelopmental disorders and setting up early interventions [13]. Yet, there are currently no shared indications of joint parent-pediatrician assessments. In addition to promoting early identification of developmental problems, an emphasis on parents as essential sources of information on children’s health offers additional benefits [14]. 

One of the factors that can impact children’s well-being is parental heightened stress [15]. Parental stress (maternal or paternal) is typically experienced when the demands of the parenting role exceed coping abilities [16, 17]. Parents with higher parenting stress levels are more likely to see their children display more deficits in language, social, and cognitive skills [18, 19]. 

Mothers’ negative emotionality is linked with unsupportive parental responses and subsequent child behavior problems [20–22]. Evidence such as this suggests that a negative maternal experience represents a risk factor for developing behavioral problems and neuropsychiatric disorders in infancy. A recent study [23] highlighted that parenting stress during infancy (11.4 ± 3.1 months) was significantly associated with mental health problems in 3-year-old children. Current views emphasize a multifactorial and multi-determined conception of parenting stress involving parent-related sources and individual distress [24–26].

However, a bidirectional association between parenting stress and child functioning has been found, of which internalizing and externalizing behaviors were the primary interests [19]. A recent study reported bidirectional associations between parenting stress and child psychopathology (with children aged 5 to 9) at different levels of maternal affection [27]. Findings of a recent systematic review [28] indicated that healthcare professionals could intervene on some modifiable factors, such as parental depression and social support, through tailored support; this might guide the development of preventive interventions and strategies.

The aim of the present study was three-fold: (a) to test the feasibility of using parent-report instruments to detect warning signs in their children’s development; (b) to ascertain whether there is an agreement between the family pediatricians’ (FP) clinical judgments of children’s warning signs and the parental perceptions; (c) to determine whether there is a link between parents’ distress and child development.

## Methods

### Participants and procedure

The Laboratory of Epidemiology of Developing Age of the Istituto di Ricerche Farmacologiche Mario Negri IRCCS in Milan set up the NASCITA Birth Cohort in collaboration with the National Pediatric Cultural Association (ACP). The methods of the NASCITA study and the baseline cohort characteristics have been described elsewhere [29, 30]. Briefly, all Italian children receive primary health care from a FP until they are six years old as part of the national health system’s organization. The population consists of infants born during the enrollment period (April 1st, 2019–July 31st, 2020) and seen by the pediatricians for seven well-child visits (within 45 days of life, at 3, 6, 12, 24, 36, and 72 months) to monitor growth and development.

**Fig. 1:**
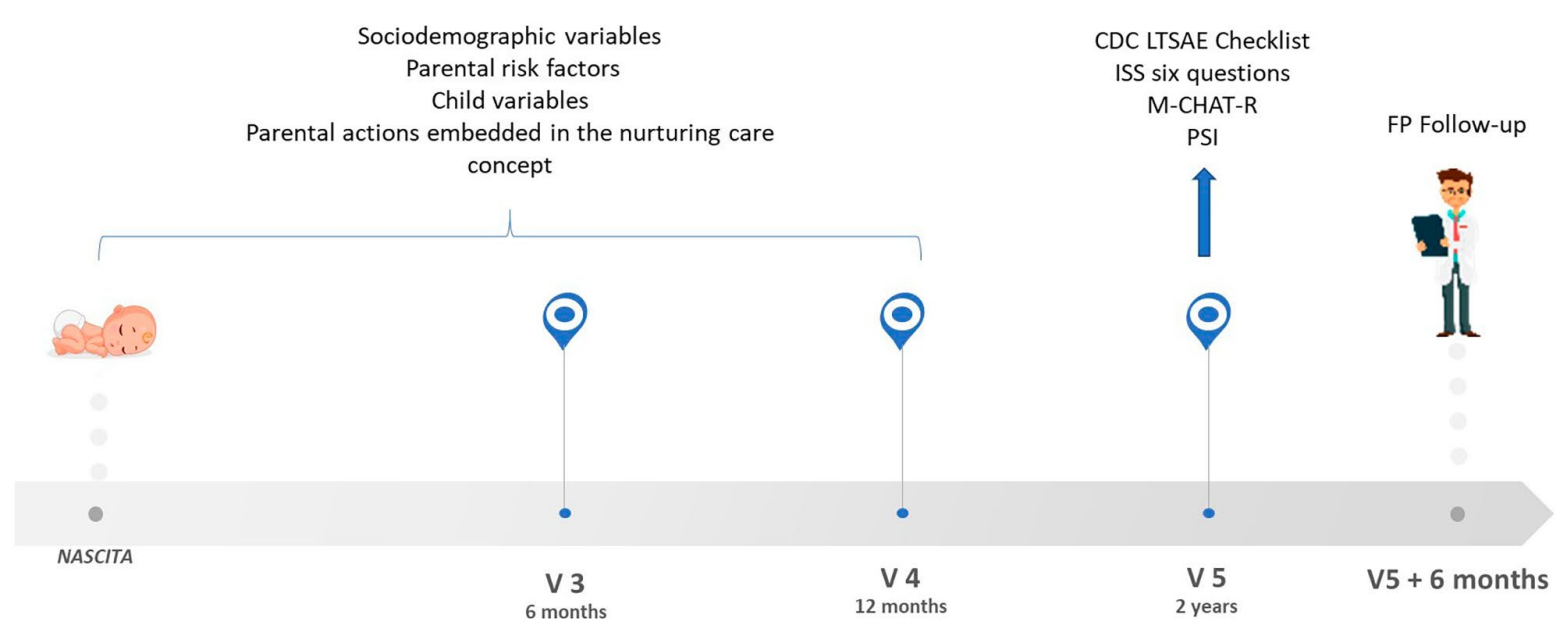
Study design and variables collected at the different time-points

The present study focuses on the assessment and the questionnaires completed at the two-year well-child visit (Fig. 1). Before starting with data collection, all pediatricians have been trained with online webinars to improve their competencies in completing the different tools. All parents gave their written consent to participate in the study. We followed the Strengthening the Reporting of Observational Studies in Epidemiology (STROBE) reporting guidelines. The study was approved by the Fondazione IRCCS Istituto Neurologico Carlo Besta’s Ethics Committee (February 6th, 2019, protocol n. 59).

### Measures

For the pediatric assessment, the FPs, in addition to the routine questions on physical growth and health care check, fill in the CDC’s Learn the Signs, Act Early Milestones (LTSAE) Checklist at two years, which was adapted from the American Academy of Pediatrics’ Bright Futures Developmental Milestones checklist. To follow the Istituto Superiore di Sanità (Italian National Institute of Health – ISS) recommendations, four questions (included in the 18–24 months checklist) were added to the CDC checklist: (1) the child walks alone, kicks the ball or other object; (2) the child gets off the ground without support; (3) the child holds a pencil or a stick and scribbles on paper or the ground/floor; (4) when the child is denied something and/or has reactions of frustration, he can usually be calmed down quickly. Moreover, two additional questions on the child’s development were included: the first one investigated the loss of competencies compared to the previous assessment, while the second one was related to the detection of hyperactivity signs by the FP.

FPs identified toddlers as “positive” for high risk if the total number of items that failed during the FP’s assessment was ≥ 8. This cutoff was defined by classifying scores in percentiles and considering the ones above the 95th percentile as a warning sign.

For the parental assessment, parents filled in The “Modified Checklist for Autism in Toddlers, Revised” (M-CHAT-R), a screening tool for the early detection of warning signs in children validated in Italian by Salomone, Cecil, and Muratori (2014) [31]. This assessment tool was chosen because some of the FPs involved in the study already used it as part of their routine clinical assessment. For this study, children were considered as having Autism Spectrum Disorder (ASD) warning signs if they fell under the medium risk category (score ≥ 3).

To follow up on the child’s development, after nearly six or more months since their visit, we contacted the FP to ask whether the child had been referred for further evaluation to a neuropsychiatrist o psychologist, as per clinical practice, and if a diagnosis was made.

Data on parental distress was also collected through the Parenting Stress Index - short form (PSI-SF) [16, 32]. Only a sub-cohort of parents voluntarily decided to fill in the questionnaire. Mothers and fathers were requested to complete the questionnaires separately; where this was impossible, we asked that at least one parent fill it in [see Appendix].

### Population profile covariates

When defining the variables to be collected, we started examining those already collected by pediatricians during the two years well-child visits. We then selected and asked pediatricians to add to their screening those variables highlighted in the literature as possible risk or protective factors for child neurodevelopment and parental stress; variables previously associated with a child presenting warning signs were selected as covariates [33–35]. The complete list of covariates is reported in Appendix 2. All collected variables were added to the statistical model in the analyses to verify their contribution as risk/protective factors in our sample.

Mothers were grouped according to their pre-pregnancy BMI into three categories: underweight (≤ 18.5), normal (18.6–24.9), and overweight or obese (≥ 25.0) [36, 37]. We also considered whether delivery happened before or after the spread of the COVID-19 pandemic. Changes in birth assistance characterized the first pandemic phase, with, for example, the impossibility of the fathers entering the delivery room [38]. In our sample, the pre-pandemic births happened between April 1st, 2019, and February 23rd, 2020, while the pandemic births occurred between February 24th, 2020, and July 31st, 2020. Type of delivery, skin-to-skin contact at birth, gender of the neonate, physiological development, and sleeping disorders were also analyzed as perinatal and postnatal variables. We also included the “healthy newborn” variable, which indicates toddlers who were not born preterm, had no malformations at birth and were not admitted to the intensive care unit in the postnatal period.

Moreover, different supportive parental actions embedded in the nurturing care concept and known to affect childhood health, development, and well-being positively were considered covariates [39–41]. They were grouped into four general intervention areas: (1) Prevention in pregnancy: no alcohol, no smoking during pregnancy; (2) Prevention after birth: exclusive breastfeeding for at least six months; (3) Approaches: Reading aloud, tummy time, bedtime routine; (4) Lifestyle: TV time in the home, screen time exposure, interacting with devices, outdoor activities (Appendix 2).

### Statistical analyses

Categorical variables were summarised using proportions and frequency distributions, and associations were tested using chi-square or chi-square for trend and Fisher’s exact test where applicable. Continuous variables were summarised using medians and interquartile ranges. Wilcoxon’s test was used to test differences in the distributions of continuous variables between two groups. Odds ratios (OR) were computed, and statistical significance was evaluated using 95% confidence intervals. The agreement between parents and pediatricians’ assessment of the same infant was calculated with Cohen’s kappa.

The sensitivity and specificity of pediatrician evaluation and M-CHAT-R in identifying children with a developmental disorder diagnosis were estimated. Logistic stepwise regression analyses were used to identify variables associated with a greater likelihood of presenting warning signs at pediatrician or parental evaluation. All variables listed in the Appendix were entered into the model and the a priori criteria of probability to enter the predictor in the model was set as less than or equal to 0.05 and for removing the predictor as greater than or equal to 0.05. We used pairwise deletion for missing data so that all variables were used. The Hosmer-Lemeshow test was used to determine the goodness of fit of the logistic regression model (*p* = .3633). Data management and analyses were performed using SAS software, version 9.4 (SAS, Institute Inc., Cary, NC, USA).

## Results

Forty-eight FPs agreed to be involved in this sub-study, and 532 infants with their parents were recruited. Data concerning the follow-up assessment were available for 435 children (232 male and 203 female) included in the present study. No further information was collected for the other 97 toddlers due to the FPs’ retirement.

FP and parental assessments were mainly different, with only six overlapping questions (i.e., the ones evaluating pretend play, climbing onto furniture, pointing, walking, copying others, and following orders). For these, the agreement between parents and pediatricians’ assessment was minimum, *k* = 0.18 (95% CI -0.04-0.31). From the M-CHAT-R, 399 children (91.7%) were classified as low risk, 33 (7.6%) as medium risk, and 3 (0.7%) as high risk for ASD or other neurodevelopmental disorders.

From either the FP assessment or the parental M-CHAT-R questionnaire, 69 toddlers (15.9%) presented warning signs. In particular, 33 children had warning signs only in the FP assessment, 26 only in the M-CHAT-R, and 10 in both assessments; 366 were not at risk. For the 69 toddlers with warning signs, associations with risk and protective factors were analyzed to identify possible markers of risk conditions (Table 1). In the univariate analysis, being male (OR 1.94, 95% CI 1.13–3.32) or having at least one foreign-born parent (OR 2.13, 95% CI 1.06–4.26) were significant risk factors. On the other hand, protective factors were reading aloud (not exposed vs. exposed: OR 2.93, 95% CI 1.72–4.97) and spending at least one hour per day outdoors (not exposed vs. exposed: OR 2.08, 95% CI 1.23–3.51). The logistic regression analysis confirmed being male (OR 2.46, 95%CI: 1.23–4.91) as a factor associated with a greater likelihood of warning signs, in addition to having sleep disorders (OR 2.43, 95% CI 1.17–5.04), while reading aloud was confirmed as a protective factor, with children not exposed versus exposed having an OR = 2.98 (95% CI 1.54–5.78).

**Table 1:** Association between child and parental characteristics associated with the presence of warning signs at parental or FP assessment

Sociodemographic variables					
		**Children at risk** (*N* = 69)	**Not at risk** (*N* = 366)	**OR (95%CI)**	*p* **-value**
Geographical area of residence	*North*	52 (75.4%)	272 (74.3%)	1.51 (0.68–3.33)	0.29^a^
	*Center*	9 (13%)	31 (8.5%)	2.29 (0.80–6.50)	
	*South*	8 (11.6%)	63 (17.2%)	1	
Both parents Italian	*Yes*	*56 (*81.2%)	330 (90.2%)	1	0.03*
	*No*	13 (18.8%)	36 (9.8%)	2.13 (1.06–4.26)	
Maternal age at delivery	*< 30*	13 (19.7%)	83 (23%)	1	0.46^a^
	*30–34*	29 (43.9%)	124 (34.3%)	1.49 (0.73–3.04)	
	*35–39*	16 (24.2%)	112 (31%)	0.91 (0.42-2)	
	*> 39*	8 (12.1%)	42 (11.6%)	1.22 (0.47–3.16)	
Paternal age at delivery	*< 30*	5 (7.6%)	46 (12.8%)	1	0.58^a^
	*30–34*	19 (28.8%)	95 (26.5%)	1.84 (0.65–5.24)	
	*35–39*	21 (31.8%)	121 (33.8%)	1.60 (0.57–4.48)	
	*> 39*	21 (31.8%)	96 (26.8%)	2.01 (0.71–5.68)	
Maternal educational level^b^	*High*	57 (82.6%)	322 (88.2%)	1	0.20
	*Low*	12 (17.4%)	43 (11.8%)	1.58 (0.78–3.17)	
Paternal educational level^b^	*High*	48 (71.6%)	295 (81.5%)	1	0.06
	*Low*	19 (28.4%)	67 (18.5%)	1.24 (0.96–3.16)	
Maternal employment status	*Employed*	48 (69.6%)	280 (76.7%)	1	0.21
	*Unemployed*	21 (30.4%)	85 (23.3%)	1.44 (0.22–2.54)	
Marital status	*With partner*	67 (97.1%)	357 (97.8%)	1	0.66
	*Single mother*	2 (2.9%)	8 (2.2%)	1.33 (0.28–6.41)	
***Parental risk factors***:					
Maternal chronic conditions	*Yes*	15 (21.7%)	88 (24%)	0.88 (0.47–1.63)	0.68
	*No*	54 (78.3%)	278 (76%)	1	
Paternal chronic conditions	*Yes*	12 (17.4%)	57 (15.6%)	1.14 (0.57–2.25)	0.71
	*No*	57 (82.6%)	308 (84.4%)	1	
Pre-pregnancy BMI	*Underweight*	4 (6%)	32 (8.8%)	0.75 (0.25–2.23)	0.24
	*Normal*	42 (62.7%)	252 (69%)	1	
	*Overweight* *or obese*	21 (31.3%)	81(22.2%)	1.56 (0.87–2.78)	
Gestational weight gain	*Below*	22 (32.8%)	132 (36.5%)	0.90 (0.49–1.65)	0.77
	*Normal*	28 (41.8%)	151 (41.7%)	1	
	*Over*	17 (25.4%)	79 (21.8%)	1.16 (0.6–2.25)	
Physiological pregnancy	*Yes*	52 (75.4%)	308 (84.2%)	1	0.08
	*No*	17 (24.6%)	58 (15.8%)	1.74 (0.94–3.21)	
Delivery during first pandemic wave^c^	*Yes*	9 (13%)	54 (14.8%)	0.87 (0.41–1.85)	0.71
	*No*	60 (87%)	312 (85.2%)	1	
***Child variables***:					
Primiparous	*Yes*	33 (47.8%)	188 (51.5%)	0.86 (0.52–1.44)	0.57
	*No*	36 (52.2%)	177 (48.5%)	1	
C-section delivery	*Yes*	24 (34.8%)	96 (26.2%)	1.50 (0.87–2.59)	0.14
	*No*	45 (65.2%)	270 (73.8%)	1	
Healthy newborn	*Yes*	57 (82.6%)	312 (85.2%)	1	0.58
	*No*	12 (17.4%)	54 (14.8%)	1.22 (0.61–2.42)	
Newborn gender	*Male*	46 (66.7%)	186 (50.8%)	1.94 (1.13–3.32)	0.02*
	*Female*	23 (33.3%)	180 (49.2%)	1	
Skin to skin contact at birth	*Yes*	48 (69.6%)	281 (77.8%)	1	0.14
	*No*	21 (30.4%)	80 (22.2%)	1.54 (0.87–2.72)	
Child sleeping disorders (from 6 months to 2 years)	*Yes*	18 (26.1%)	74 (20.2%)	1.39 (0.77–2.52)	0.27
	*No*	51 (73.9%)	292 (79.8%)	1	
***Parental actions embedded in the nurturing care concept: prevention in pregnancy***					
Mother smoker in pregnancy	*Yes*	8 (11.6%)	23 (6.3%)	1.94 (0.83–4.55)	0.13
	*No*	61 (88.4%)	341 (93.7%)	1	
Mother consuming alcohol in pregnancy	*Yes*	8 (11.6%)	43 (11.9%)	0.97 (0.43–2.17)	0.94
	*No*	*61 (88.4%)*	*318 (88.1%)*	1	
***Parental actions embedded in the nurturing care concept: prevention after birth***					
Exclusive breastfeeding for at least 6 months	*Yes*	22 (32.8%)	119 (33.1%)	1	0.96
	*No*	45 (67.2%)	240 (66.9%)	1.01 (0.58–1.77)	
***Parental actions embedded in the nurturing care concept: Approaches***					
Reading aloud to children	*Yes*	27 (39.1%)	239 (65.3%)	1	< 0.001***
	*No*	42 (60.9%)	127 (34.7%)	2.93 (1.72–4.97)	
Tummy time	*Yes*	53 (76.8%)	312 (86.2%)	1	0.05
	*No*	16 (23.2%)	50 (13.8%)	1.88 (1-3.55)	
Bedtime routine	*Yes*	13 (21.7%)	87 (26.9%)	1	0.39
	*No*	47 (78.3%)	236 (73.1%)	1.33 (0.69–2.58)	
***Parental actions embedded in the nurturing care concept: Lifestyle***					
Outdoor activities	*Yes*	30 (44.1%)	220 (62.1%)	1	< 0.01**
	*No*	38 (55.9)	134 (37.9%)	2.08 (1.23–3.51)	
Screen exposure^d^	*Yes*	16 (23.2%)	97 (26.6%)	1	0.55
	*No*	53 (76.8%)	267 (73.4%)	1.20 (0.66–2.20)	
Interacting with devices	*Yes*	26 (40%)	142 (39.7%)	1	0.96
	*No*	39 (60%)	216 (60.3%)	0.99 (0.57–1.69)	
TV-on time in the home	*Yes*	49 (74.2%)	260 (76.5%)	1	0.70
	*No*	17 (25.8%)	80 (23.5%)	1.13 (0.62–2.07)	

^*a*^ *p-value of chi-square for trend test.* ^*b*^ *Educational level: low: no schooling or primary versus high: secondary school or university.* ^c^ *Delivery during first pandemic wave: Yes: delivery between 24/02/2020 and 31/07/2020; No: delivery between 01/04/2019 and 23/02/2020.* ^*d*^ *Screen exposure yes refers to the adoption of a positive parental approach. See Appendix* 2 *for the definition of the variables*

Significant p values are marked as follows: *p < .05, two-tailed. **p < .01, two-tailed. **p < .*001, two-tailed*

*Missing data were not reported in the table because they were absent or limited to < 2.5% for all the covariates, except TV on time in the home (missing: 29/435) and bedtime routine (missing: 52/435). For these latter variables, the distribution of missing data in the 2 groups was similar*

Follow-up revealed that among the 69 toddlers with warning signs, 26 were referred for further evaluation to a neuropsychiatrist or psychologist. In addition, 7 of the toddlers who did not present warning signs were also referred. Out of 33 children who underwent a specific clinical evaluation, 16 received a diagnosis (of whom 14 had warning signs and 2 did not): language disorders (*n* = 9), autism (*n* = 3), developmental delays (*n* = 2), and other conditions (*n* = 2). For the M-CHAT-R, a sensitivity of 62.5% (95% CI 35.4–84.8%) and a specificity of 76.5% (95% CI 50.1–93.2%) were calculated. For the FPs’ assessments, a sensitivity of 75.0% (95% CI 47.6–92.7%) and a specificity of 41.2% (95% CI 39.7–75.0%) were estimated. Combining the M-CHAT and FP assessments, we observed a sensitivity of 87.5% (61.7–98.4%) and a specificity of 29.4% (95% CI 10.3–56.0%).

### Parental stress

At least one parent completed the PSI questionnaire for 397 children (61 with warning signs). Seventy-three children (18.4%) had one parent (mother or father) whose distress was considered clinically significant (≥ 85) in the total score. Nine children (5.4%) had both parents clinically distressed. The prevalence of parental distress was 14.9% among mothers (*n* = 368) and 13.8% among fathers (*n* = 195).

Among the 61 children considered at-risk by the parental or pediatrician assessments, 19 (31.1%) had at least one parent whose distress was significant in the PSI. The presence of warning signs in children (either in the FP or parental assessment) and parental distress (OR 2.36, 95% CI 1.27–4.37) were positively associated. This association was confirmed with an increased prevalence of maternal (OR 2.42, 95% CI 1.23–4.76) but not paternal distress. The period of birth (pre-pandemic vs. first wave) did not influence the likelihood of developing warning signs or parental stress. Of the children referred for further evaluation, 9 (35%) had at least one distressed parent, and of those who received a diagnosis, 4 (25%) had at least one distressed parent.

## Discussion

The follow-up assessment was completed for 435 infants, of whom 69 (15.8%) presented warning signs. Being male and having sleep disorders was associated with a greater likelihood of warning signs in the multivariate analysis, while reading aloud was a protective factor. For 73 children (18.4%), at least one parent tested positive for PSI-SF; an increased prevalence of parental distress was observed in children with warning signs.

Although a few recent studies have highlighted the importance of a shared approach between parents and pediatricians in screening and encouraging early diagnoses [7, 42–44], the present study is the first to integrate the parental and FP perspectives to detect those children with possible warning signs early. The feasibility of integrating the FPs and parental perspectives was confirmed. In our cohort, 1 out of 6 children was considered “at risk” at the FP assessment (10.1%), the parental M-CHAT-R questionnaire (8.3%), or both. Similar percentages were found in several international studies that used the CDC [45] or the M-CHAT-R questionnaire [46–49]. The combined risk percentage assessed in this study was indeed higher, precisely 16%. The pediatricians, however, did not consider a psychiatric or psychological evaluation essential for most of these children. Because approximately 1 out of 20 children were referred for further evaluation, this proportion was consistent with previous literature [47]. 

Only for 10 out of 69 children the warning signs were reported by both parents and FPs. This number was expected since the tools (M-CHAT-R and CDC checklist) evaluate different skills and domains. It should be underlined, however, that the agreement between parents and pediatricians was low when considering the six overlapping questions in the M-CHAT-R and the CDC questionnaires (considering pretend play, climbing onto furniture, pointing, walking, copying others, and following orders). This observation converges with the idea that parents and FPs may have different perceptions of the warning signs and that a shared approach is needed, as documented by the increase in sensitivity when combining the two evaluations.

Consistent with the findings of other studies [50–52], signs of potential neurodevelopmental disorders resulted more frequently in males. An Indonesian study [53] used the M-CHAT-R to screen children aged 16–30 months and showed that 3.5% of the subjects were at high risk of developing ASD; only the male gender was significantly associated with ASD. The male-to-female ratio with moderate-to-high-risk M-CHAT-R results was 2.5:1. Similarly, a male-female ratio of 2:1 exists among individuals with intellectual disability/developmental delay [54] and a 4:1 ratio for individuals with autism diagnoses [55]. Moreover, an association between sleep disorders in children and developmental disorders, such as pervasive developmental disorder (PDD) and attention deficit hyperactivity disorder (ADHD), was detected [56, 57]. Having at least one foreign parent was also associated with an increased likelihood of having warning signs. Some aspects, in particular socio-demographic variables (e.g., parental level of education, and parental nationality) could indicate other significant factors that might directly impact on children warning signs and development. Parental race, ethnicity, and culture can affect child development. Parent-child relationships and parental behavior affect children’s health and well-being across ethnicities, cultures, and genders in traditional as well as nontraditional families. Present findings are consistent with reported more specific and appropriate evaluations, suggesting an in-depth analysis in future studies [58, 59]. On the other hand, in the present study reading aloud and spending at least one hour per day outdoors were protective factors: parents whose infants had a lower likelihood of warning signs were more adherent to those actions embedded in the nurturing care concept. Previous studies describe both these factors as having a protective role in neurodevelopment [60, 61].

From the parental assessment, almost one out of five children was observed to have one parent with clinically significant distress. Considering the children “at-risk,” this percentage rose to nearly one-third. The prevalence of parental distress was similar in mothers and fathers. In the literature, parents of a non-clinical sample reported a lower prevalence of stress, i.e., between 7 and 9% [62, 63]. It is important to highlight that no recent studies report updated estimates. Therefore, several factors (e.g., the COVID-19 pandemic, financial difficulties, and the environmental crisis) might have determined increased stress levels. Fewer studies involving parents of children and adolescents with neurodevelopmental disorders have reported high-stress levels in their parental role [64]. An association was also found between the presence of warning signs in infants and parental stress in mothers, while the same was not confirmed in fathers. The same gender difference was previously reported when evaluating parents of children with other clinical conditions [65]. Research has shown that the relationship between child behavioral problems and parental stress is bidirectional [66]. Since parental stress data have been collected only when child was 24 months, it is impossible to verify if it is antecedent or consequent to the child’s difficulties. It has been tested whether there was a difference in parental stress considering delivery child in pandemic times but, although literature shows that there was an increase in parental stress during pandemic time in Italy [67] the results if the present study found no differences at 24 months visit between parents of children born before or after pandemic time. Parents with poorer psychological well-being may perceive their child’s behavior more negatively. At the same time, parental distress may interfere with the parent’s ability to remain sensitive and accepting of the child [68]. Those children might, therefore, receive less protective actions such as reading aloud and spending time outdoors.

Some limitations must be considered. Even if neurodevelopmental disorders may be recognized at 18 months, the assessment was scheduled at 24 months consistently with the timing of well-child visits in most Italian regions. Therefore, in a few cases, the warning signs may have been present earlier. Moreover, the FPs involved participated voluntarily and might not be fully representative of all Italian family pediatricians. The COVID-19 pandemic represented a challenge for the NASCITA cohort study and may have impacted parents’ and children’s well-being. We included the period of birth (pre-pandemic vs. first wave) among the covariates, which did not influence the likelihood of developing warning signs or parental stress.

Nonetheless, we cannot guarantee that the stressful situation that families had to face in the years after the children’s birth did not contribute to increasing parental stress and even affected child at-risk situations. The parental distress has been evaluated with the PSI-SF, one of the instruments commonly used as a diagnostic or screening measure to assess the parenting system and possibly identify at-risk or problematic areas in parental behavior. In the present study, parents were asked to fill it in as a self-report questionnaire that the FPs then collected. This tool per se cannot constitute the basis for a clinical diagnosis. Still, those who emerged as potentially at risk could be advised to pursue a specific clinical assessment (which cannot be done in the context of well-child pediatric visits).

An important consideration is that even though referral to a specialist when needed is foreseen in clinical practice, only 38% of the children with warning signs have been evaluated by a child psychiatrist or a psychologist. In some cases, a diagnostic process might still be ongoing. Thus, the number of children with clinical diagnoses reported in the present study may be underestimated. It is possible that FPs decided to refer only children with a suspected developmental disorder and less severe warning signs required only a follow-up by the FPs. Lastly, in Italy, mothers are the caregivers who more frequently bring their children to the well-child visits; reduced paternal involvement in the present study confirmed this assumption. Greater paternal involvement in monitoring a child’s development should be encouraged. Therefore, it will be necessary to monitor the development of this cohort in future years to confirm the accuracy of the proposed assessment model.

This study demonstrated the feasibility of a new approach: combining the results of parental perspective and pediatrician’s evaluation could help identify children at risk of neurodevelopmental disorders early. Thus, the present study does lay the foundation for future studies on early screening during well-child visits. Moreover, its longitudinal design permits overcoming the large variability of the developmental trajectory at early stages: children should be followed up in terms of development at different time points regardless of screening status; repeated developmental assessments over time are more informative than one-time assessments in planning investigations and management. Pediatricians should also consider parental emotions, especially depression, and stress, which, as demonstrated, could be evaluated with self-report questionnaires and could have a potential association with children’s outcomes.

Hopefully, this study will help bridge the gap between screening tools and a practical protocol that can be applied in the FP’s setting, enhancing the capability for early identification of children who appear to be at low risk of a developmental disorder. Further scientific studies with more sophisticated designs are needed to better understand the different aspects of possible developmental disorders and associated factors; moreover, future research is required to ensure that developmental interventions are effectively conducted on children at risk and referred to specialists. An ongoing relationship between pediatricians and parents will guarantee better neurodevelopmental outcomes.

## Conclusions

The present study demonstrated the feasibility and usefulness of child assessment that integrates the FPs’ and parents’ perspectives and evaluations: future studies might implement a similar approach, asking parents to fill in different questionnaires (as the MCHAT used here) to report on their child’s development. Secondly, while the agreement between parents and FPs was only minimum, the combined sensitivity was higher. Thus, monitoring the infant’s development in different contexts is crucial and should always be recommended. Future directions should follow up with children and parents at later stages, possibly involving other figures with a primary role in children’s development, such as educators and teachers. Finally, this study highlights variables that could significantly impact both child development and parental distress. Valuable practical interventions designed around these factors could be implemented and used by the FPs to support parents. Where significant stress is observed, parents might benefit from information on positive behaviors (i.e., supportive parental actions) to lower perceived stress and support a child’s development. Where this is not enough, FPs can recommend clinical follow-ups.

### Electronic supplementary material

Below is the link to the electronic supplementary material.

Supplementary Material 1

## Data Availability

The datasets used and/or analyzed during the current study are available from the corresponding author upon reasonable request.

## Abbreviations

NASCITA: (NAscere e creSCere in ITAlia)
M-CHAT-R: Modified Checklist for Autism in Toddlers, Revised
PSI-SF: Parenting Stress Index, Short Form
OR: Odds Ratio
CI: Confidence Interval
FP: Family Pediatricians
LTSAE: Learn the Signs. Act Early
ISS: Italian National Institute of Health
ASD: Autism Spectrum Disorder
BMI: Body Mass Index
COVID-19: Coronavirus Disease-19
NICU: Neonatal Intensive Care Unit
CDC: Centers for Disease Control and Prevention
PDD: Pervasive Developmental Disorder
ADHD: Attention-Deficit / Hyperactivity Disorder
