# Supplementary material for: Pediatrician and parental evaluation of child neurodevelopment at 2 years of age
Source: BMC Pediatr. 2024 Feb 22;24:137. doi: 10.1186/s12887-024-04616-2 (PMC10882900; doi:10.1186/s12887-024-04616-2)
Supplement: Supplementary file 1 — Supplementary Material 1 [file 12887_2024_4616_MOESM1_ESM.docx]

**Pediatrician and parental evaluation of child neurodevelopment at 2 years of age**

**Appendix**

Giulia Segre^a^, Antonio Clavenna ^a^, Elisa Roberti ^a^, Francesca Scarpellini ^a^, Massimo Cartabia^a^, Chiara Pandolfini^a^, Valeria Tessarollo^b^, Ilaria Costantino^b^ and Maurizio Bonati^a*^

**Affiliations:** ^a^ Laboratory of Epidemiology of Developing Age, Department of Medical Epidemiology, Istituto di Ricerche Farmacologiche Mario Negri IRCCS, Milan, Italy and ^b^ Child Neuropsychiatry Unit, Department of Health Sciences, ASST Santi Paolo e Carlo, San Paolo Hospital, Università degli Studi di Milano, Milan, Italy

**^*^Address correspondence to:** Maurizio Bonati, Laboratory of Epidemiology of Developing Age, Department of Medical Epidemiology, Istituto di Ricerche Farmacologiche Mario Negri IRCCS. Via Mario Negri 2, 20156, Milan (Italy). [maurizio.bonati@marionegri.it](mailto:maurizio.bonati@marionegri.it). Phone: 0039 02 390141.

***APPENDIX 1***

*FPs assessment:*

The CDC assesses the child's language, cognitive/adaptive, motor, and social-emotional skills development (CDC- Developmental Milestones 2019, Bright Future 2020).[1] This checklist is an evidence-informed developmental surveillance tool to identify early children with developmental delays and disabilities. The original CDC was translated from English into Italian. A back-translation was then performed by bilingual specialists who had never seen the original CDC. Three developmental and behavioral pediatricians then examined the Italian version. The back-translated versions were then compared with the original English version to assess the accuracy and resolve any discrepancies, as recommended by Jones, Lee, and Phillips (2001). [2]

To follow the Istituto Superiore di Sanità (Italian National Institute of Health – ISS) recommendations, four questions (included in the 18-24 months checklist) were added to the CDC checklist: 1) the child walks alone, kicks the ball or other object; 2) the child gets off the ground without support; 3) the child holds a pencil or a stick and scribbles on paper or the ground/floor; 4) when the child is denied something and/or has reactions of frustration, he can usually be calmed down quickly. Moreover, two additional questions on the child's development were included: the first one investigated the loss of competencies compared to the previous assessment, while the second one was related to the detection of hyperactivity signs by the FP.

*Parental assessment:*

The “Modified Checklist for Autism in Toddlers, Revised” (M-CHAT-R) is a measure developed to assess the risk for ASD in children ages 16-30 months. The M-CHAT-R is a simple screening tool considered valid for the early detection of warning signs in children compared to the conventional method (Italian translation: Salomone, Cecil, and Muratori, 2014).[3] It is a checklist of 20 commonly observed child behaviors that requires the parent to report the presence of each specific behavior with a yes/no response. There are no culturally biased items. For all items except questions 2, 5, and 12, the response "NO" indicates a warning sign. The presence of abnormal behavior is assigned a score of 1, and the total score (obtained through the sum of all items where the response is identified as a warning sign) is interpreted. A total score of ≤2 indicates a low risk for ASD, and no further follow-up is recommended. When a total score between 3 and 7 is obtained, second-stage follow-up questions should be administered to decrease false-positive results: they were reassessed using M-CHAT-R Follow up questionnaire only on those items that originally failed. Children who were found to be in the low to medium-risk category were advised to have regular follow-ups. When a score of ≥8 is obtained, bypassing follow-up and immediately referring for diagnostic evaluation is allowed.

*Parental distress:*

Data on parental distress were collected with the Parenting Stress Index - short form (PSI-SF). [4,5] The PSI-SF consists of 36 items measuring stress levels within the parent-child relationship. The respondent is asked to answer each item on a five-point Likert scale, ranging from 1 (strongly agree) to 5 (strongly disagree). The questionnaire yields a Total Stress score from three subscales. The Parental Distress subscale (PD; e.g., "I feel trapped by my responsibilities as a parent") taps the parent's perception of their behavior, including, for instance, perceived competence and sacrifices because of their parenting role. The Parent-Child Dysfunctional Interaction subscale (P-CDI; e.g., "My child rarely does things for me that make me feel good") refers to the parent's judgments on the interaction with their child. Finally, the Difficult Child subscale (DC; e.g., "My child makes more demands on me than most children") measures the parent's perception of his/her child's personality, demandingness, hostility, and obedience. The Italian version of the PSI-SF[5] has shown good internal consistencies ranging from α=.95 for the P-CDI subscale to α=.90 for the DC subscale. In the present study, for mothers, the internal consistency of the PSI-SF corresponds to α=.91 for the total stress scale. For fathers, the internal consistency of the PSI-SF corresponds to α=.92 for the total stress scale. A higher score suggests a higher stress level. Parenting stress percentile scores that fall between 15 and 84 are considered typical; scores above 85 indicate (at the 90th percentile) clinically significant parental stress. The total stress score (TS) is obtained by adding the scores of the three subscales PD, PCD-I, and DC. The test also includes a defensive response scale (DF) to check the validity of the protocol as it indicates whether the parent tends, for example, to give a better self-image or to minimize problems and perceived stress in the relationship with the child.

***APPENDIX 2***

Variables that in previous studies have been associated with the presence of child warning signs (at parental or FP assessment) were selected as covariates. The complete list of covariates is reported in Table 1.

Sociodemographic variables:

- geographical area of residence: North, Centre and South of Italy;
- having parents who are both Italian;
- parents' age at delivery;
- parental educational level;
- maternal employment status;
- maternal marital status.

Parental risk factors:

- parental chronic conditions;
- maternal pre-pregnancy BMI;
- maternal gestational weight gain;
- physiological pregnancy;
- delivery during first pandemic wave (24/02/2020- 31/07/2020) vs pre-pandemic wave (01/04/2019-23/02/2020).

Child variables:

- first-born child;
- C-section delivery (yes = instrumental vs no = spontaneous);
- healthy newborn: children in this cathegory were born at term, without malformations and were not admitted to NICU at birth;
- gender of the neonate;
- skin-to-skin contact at birth;
- child sleeping disorders from 6 months to 24 months.

Parental actions embedded in the nurturing care concept in pregnancy:

- mother smoker in pregnancy;
- mother consuming alcohol in pregnancy.

Parental actions embedded in the nurturing care concept after birth:

- exclusive breastfeeding at six months.

*Parental actions embedded in the nurturing care concept: approaches:*

- reading aloud to children: considered positive if a book had been read to the child in the two weeks prior to each of the three visits (6 months, 1 year and 2 year);
- tummy time: considered adequate if the child was placed belly down for any amount of time each day at 6 months. The American Academy of Pediatrics does not specify a precise amount of time a day, indicating a general 3-5 minutes, two to three times a day, increasing the time if the child enjoys it, for infants.[6] The WHO guidelines recommend at least 30 minutes each day in a prone position while awake for 0-1 year-olds;[7]);
- bedtime routine: this was considered positive if there was a routine and it included a book or a song, and negative if it included tv/screen or "other", at 1 and 2 year visit.[8,9] We realize that classifying "other" as negative is probably a bit stringent, but we considered the American Academy of Pediatrics' suggestions for healthy sleep habits for children. [8,10]

Parental actions embedded in the nurturing care concept: lifestyle:

- outdoor activities: At the 6 month, and 1 and 2 year visits, the parent was asked to quantify time spent by the child outdoors (1 = rarely, 2 = less than 1 hour per day, 3 = 1-3 hours per day, 4 = > 3 hours per day). If the sum of the scores was >8 it was considered adequate, if ≤ 8 it was considered insufficient. Although no precise minimum amount of time outdoors to achieve health benefits in young children has been found, at least an hour a day at one year of age was considered adequate; [14–16]
- screen exposure is considered positive (=yes) if parents do not allow children to watch videos on screens (TVs, tablets, and smartphones) at 1 and 2 years;
- interacting with devices: At both the 1 and 2 year visits, the parent was asked to indicate whether the child interacts with smartphone/tablet: (1 = never, 2 = sometimes, 3 = often). If the total score was 2 (never at both visits) the use was considered appropriate, if > 2 it was considered excessive;
- TV-on time in the home: considered adequate if ≤4 hours per day at 1 and 2 year of age (Note: the reasoning behind this factor, and the cutoff time, was that, although a TV being on in the home will likely expose the child to a certain extent, the 4-hour cutoff allowed additional time for the parents' watching of the news and of the TV when the children were in bed);[11] the child was never given a smartphone or tablet to hold, and was never or almost never allowed to watch videos on any screen at 1 year of age. [12,13]

**References**

[1] CDC’s Developmental Milestones 2019.

[2] Jones PS, Lee JW, Phillips LR, Zhang XE, Jaceldo KB. An Adaptation of Brislin???s Translation Model for Cross-cultural Research: Nursing Research 2001;50:300–4. https://doi.org/10.1097/00006199-200109000-00008.

[3] Robins DL, Barton M, Fein D. Modified Checklist for Autism in Toddlers, Revised with Follow-up 2018. https://doi.org/10.1037/t67574-000.

[4] Abidin RR. Parenting stress index. Psychological Assessment Resources, Inc. (3rd ed.). Florida: Odessa.: 1995.

[5] Guarino A, Di Blasio P, D’Alessio M, Camisasca E, Serantoni M. Parenting Stress Index Short Form. Italian Validation. Florence: Giunti O.S. Organizzazioni Speciali.; 2008.

[6] Back to Sleep, Tummy to Play. HealthyChildrenOrg n.d. https://www.healthychildren.org/English/ages-stages/baby/sleep/Pages/Back-to-Sleep-Tummy-to-Play.aspx (accessed June 8, 2022).

[7] World Health Organization. Guidelines on physical activity, sedentary behaviour and sleep for children under 5 years of age. Geneva: World Health Organization; 2019.

[8] Toddler Bedtime Trouble: Tips for Parents. HealthyChildrenOrg n.d. https://www.healthychildren.org/English/healthy-living/sleep/Pages/Bedtime-Trouble.aspx (accessed June 8, 2022).

[9] Janssen X, Martin A, Hughes AR, Hill CM, Kotronoulas G, Hesketh KR. Associations of screen time, sedentary time and physical activity with sleep in under 5s: A systematic review and meta-analysis. Sleep Med Rev 2020;49:101226. https://doi.org/10.1016/j.smrv.2019.101226.

[10] Brush, Book, Bed: How to Structure Your Child’s Nighttime Routine. HealthyChildrenOrg n.d. https://www.healthychildren.org/English/healthy-living/oral-health/Pages/Brush-Book-Bed.aspx (accessed June 14, 2022).

[11] Why to Avoid TV for Infants & Toddlers. HealthyChildrenOrg n.d. https://www.healthychildren.org/English/family-life/Media/Pages/Why-to-Avoid-TV-Before-Age-2.aspx (accessed June 8, 2022).

[12] Madigan S, Browne D, Racine N, Mori C, Tough S. Association Between Screen Time and Children’s Performance on a Developmental Screening Test. JAMA Pediatr 2019;173:244. https://doi.org/10.1001/jamapediatrics.2018.5056.

[13] Zhao J, Yu Z, Sun X, Wu S, Zhang J, Zhang D, et al. Association Between Screen Time Trajectory and Early Childhood Development in Children in China. JAMA Pediatr 2022. https://doi.org/10.1001/jamapediatrics.2022.1630.

[14] Playing Outside: Why It’s Important for Kids. HealthyChildrenOrg n.d. https://www.healthychildren.org/English/family-life/power-of-play/Pages/playing-outside-why-its-important-for-kids.aspx (accessed June 8, 2022).

[15] CFOC Standards Database | National Resource Center n.d. https://nrckids.org/CFOC/Database/3.1.3.1 (accessed June 8, 2022).

[16] Toffol G, Reali L. Il benessere psico-fisico dei bambini migliora se frequentano spazi verdi. Quaderni ACP 2018;25:1–3.
